# Intention to Exclusively Breastfeed Is Associated with Lower Rates of Cesarean Section for Nonmedical Reasons in a Cohort of Mothers in Vietnam

**DOI:** 10.3390/ijerph19020884

**Published:** 2022-01-13

**Authors:** Doan Thi Thuy Duong, Colin Binns, Andy Lee, Yun Zhao, Ngoc Minh Pham, Dinh Thi Phuong Hoa, Bui Thi Thu Ha

**Affiliations:** 1 Faculty of Social Sciences, Behavior and Health Education, Hanoi University of Public Health, 1A Duc Thang Street, Bac Tu Liem District, Hanoi 10000, Vietnam; dttd@huph.edu.vn (D.T.T.D.); bth@huph.edu.vn (B.T.T.H.); 2 School of Population Health, Curtin University, Bentley, WA 6102, Australia; Andy.Lee@curtin.edu.au (A.L.); Y.Zhao@exchange.curtin.edu.au (Y.Z.); minh.pn@tnu.edu.vn (N.M.P.); 3 Department of Epidemiology, Faculty of Public Health, Thai Nguyen University of Medicine and Pharmacy, Thai Nguyen 250000, Vietnam

**Keywords:** vaginal delivery, mode of birth, cesarean section, breastfeeding intention, Vietnam

## Abstract

Background: Breastfeeding brings benefits to both mothers and children in the short term and long term. Unnecessary cesarean sections can bring risks to both parties. This study was undertaken to examine the relationship between exclusive breastfeeding intention and cesarean delivery. Methods: We analyzed data collected from 554 single mothers who delivered in Dong Anh General District Hospital or Hanoi Obstetrics and Gynecology Hospital, Vietnam, in 2020–2021. The relationship between exclusive breastfeeding intention and cesarean delivery for nonmedical reasons was adjusted for maternal education, maternal age, parity, history of fetal loss, having at least eight antenatal contacts, hospital of delivery, child sex, and birth weight. Results: Antenatally, 34.8% (184/529) of mothers intended to breastfeed exclusively until 6 months and 30.8% (84/274) underwent cesarean section for a nonmedical reason. After adjusting for other factors, mothers who intended to breastfeed exclusively until 6 months were less likely to undergo cesarean delivery for nonmedical reasons (OR = 0.55, 95% CI: 0.31–0.96, *p* = 0.034). Conclusions: This study adds to the growing evidence related to unnecessary cesarean sections and routine over-medicalization of normal birth in the urban areas of Vietnam. The association between breastfeeding intentions and a lower rate of cesarean section suggests that education on breastfeeding could be a useful intervention for reducing the rate of cesarean sections and improving maternal and child health.

## 1. Introduction

Cesarean section is an essential and lifesaving surgery for women if vaginal delivery would pose a significant risk. However, the World Health Organization (WHO) recommended that at the population level, a rate of cesarean section exceeding 10% would not reduce maternal and newborn mortality [1]. Cesarean sections undertaken without a medical indication can put both women and children at unnecessary risk of short- and long-term health problems. To mothers, adverse health outcomes of high rates cesarean section have been reported including maternal mortality, admission to intensive care unit [ICU], blood transfusion, hysterectomy, or internal iliac artery ligation [2]. Compared with vaginal delivery, newborn infants who were delivered by cesarean section were more likely to be admitted to a neonatal intensive care unit [3], develop respiratory tract infections, and later in life, asthma, and obesity [4]. Beyond direct health effects, high rates of unnecessary cesarean sections can remove resources away from other services in overloaded and poorly resourced health systems [5].

While breastfeeding has many benefits for both mothers and children in the short term and long term [6,7], cesarean section has negative effects on breastfeeding outcomes [8,9,10]. Compared with vaginal delivery, women who had cesarean section had no intention to breastfeed or did not initiate breastfeeding, had a higher proportion of breastfeeding difficulties, and were more likely to discontinue breastfeeding before 12 weeks postpartum [8]. Rates of early breastfeeding were 43% lower after cesarean section [9]. In Vietnam, mothers who underwent caesarean section were more likely to give prelacteal feeds to their infants, had lower rates of early initiation of breastfeeding, and reduced likelihood of (any, predominant and exclusive) breastfeeding from discharge to 6 months after delivery. After 1 year, any breastfeeding rate was still lower in the caesarean delivery group [10].

Despite negative effects, the rate of cesarean section worldwide is at 21.1% and is continuing to increase [11]. The highest cesarean section rate is in Latin America and the Caribbean region (42.8%), followed by Eastern Asia (33.7%) [11]. The major driving forces behind the rising cesarean delivery trends worldwide were the preferences of mothers, families, and health professionals [12]. Choosing cesarean delivery is an interactive process of fear of the unknown or previous experience of childbirth, family, friends, the media, and healthcare professionals [13].

In urban areas in Vietnam, the cesarean section rate has reached a high level of 42.4–57.9% in public hospitals [14,15]. It is much higher than the overall cesarean section rate of 15.9% in South-East Asia [11]. The high rates of cesarean delivery [14,15,16,17,18], the medical indications [15,18], and associated sociodemographic factors [14,15,16,17,18,19] have been reported in previous studies. Adverse effects of cesarean delivery including poor breastfeeding outcomes [10] and childhood overweight [20] have also been reported. Given that the high rates of cesarean delivery are often for nonmedical reasons and not driven by sociodemographic factors [5], it is a challenge to address the recent rise in unnecessary cesarean deliveries in Vietnam and other Asian lower-middle-income countries (LMIC).

Most of the recent evidence is on the risk factors of cesarean section and its adverse effects on breastfeeding, as well as maternal and child health outcomes, as mentioned above. There are only two studies that have suggested breastfeeding intention could be a possible protective factor of having cesarean section by choice, in Alberta and Canada [8,9]. However, the difference between breastfeeding intention and cesarean section was not at a statistically significant level in another study in England [21]. If increasing breastfeeding intention can also reduce cesarean section for nonmedical reasons, mothers and children would both benefit. The relationship between breastfeeding intention and having cesarean section, therefore, requires further study. In this study, we used data collected from Vietnam to examine the association between breastfeeding intention and cesarean section.

## 2. Materials and Methods

### 2.1. Study Design

The subset of data used for analysis could be considered as a prospective cohort study, as we selected only mothers in a control group from the randomized controlled trial. The information on breastfeeding intention were collected during the prenatal period. The outcome, mode of delivery, was collected after delivery. The study population and data collection process are described in detail below.

### 2.2. Study Population

Mothers were invited to participate in a maternal care randomized controlled trial of a health promotion intervention conducted between 2019 and 2021 in Hanoi, the capital of Vietnam. The research protocol has been reported previously [22]. This paper reports the findings on the mothers forming the control group of that trial. Hanoi is amongst the fastest-growing cities in the world in terms of gross domestic product (GDP), with a population estimated to be 8 million in 2019 [23]. About 85% of people in Hanoi own a smartphone according to a report on the global mobile market in 2020. Cesarean sections are performed in public tertiary hospitals, general semiurban district hospitals, and private hospitals.

The study recruited single mothers at 24–36 weeks of gestation when they visited the two public hospitals: either Dong Anh General District Hospital or Hanoi Obstetrics and Gynecology Hospital for antenatal care (ANC). Dong Anh general district hospital is one of 17 semiurban districts in Hanoi which has 6000 deliveries per year and a cesarean section rate of 41%. Hanoi Obstetrics and Gynecology Hospital is a specialist hospital in obstetrics and gynecology in the northern region, and is mainly for people who are living in the city. In 2019, there were 44,000 deliveries and the cesarean section rate was 53%. Mothers who were classified as high-risk pregnancies or when the pregnancy resulted in a stillbirth, were excluded at baseline interview or during the follow up.

### 2.3. Data Collection

Data were collected at enrolment in the study and a week after discharge from the hospital. Eligible mothers were interviewed face-to-face when they visited the two hospitals for antenatal care. After completing a standardized questionnaire including socioeconomic variables, demography (maternal age, parity), and breastfeeding intention, half of them (633) were randomized into the control group. The control group received normal care and had no contact with the intervention program. After birth, we contacted and interviewed mothers by telephone. A total of 554 mothers in the control group completed both the baseline and hospital discharge surveys and were included in the analysis. The follow–up rate was at an acceptable level of 87.5% [24]. Reasons for loss to follow-up included wrong or unavailable phone number—4.9%, the mother declined to continue or was too busy—7.1%, and stillbirth or infant died—0.5%. The loss to follow-up rate and reasons were similar to previous phone surveys among mothers after 10 and 45 days after discharge from the hospitals in Vietnam [25].

A group of 10 research assistants, who were not working in the hospitals, conducted the interviews after obtaining consent forms from mothers. The mode of delivery and sex of the baby were confirmed using hospital medical records.

#### 2.3.1. Dependent Variables

The outcome of interest was the mode of delivery (vaginal or cesarean section) based on maternal self-reported response to the question “How was your baby delivered?”. Responses were classified into vaginal and cesarean section delivery. If mothers had cesarean section delivery, we asked “what is the main reason for cesarean section?”. The answers were recorded as medical reasons, nonmedical reasons (doctor’s suggestion, family’s preference, fear of delivery pain, and more convenient to choose the birth date), and unknown. Medical reasons were coded following ICD-10 for indication of cesarean section into nine groups: hypertensive disorder, malpresentation, a disorder of amniotic fluid, antepartum hemorrhage including placenta previa, post-dated pregnancy, prolonged and obstructed labor, fetal distress, previous cesarean delivery, a maternal disorder related to pregnancy, and general disease complicating pregnancy.

#### 2.3.2. Independent Variables

Intention to breastfeed exclusively until 6 months was the variable of interest of this study and was based upon self-reports by pregnant mothers in response to three questions: “What will you feed your child during the first 6 months?”, “When will you feed your child with complementary food?”, and “when will you feed your child with liquids including water?” as the baseline survey. Mothers who planned to feed their child only with breastmilk without giving any complementary foods or liquid until 6 months were coded as having exclusive breastfeeding intention following the WHO definition [26].

We selected other potential covariates purposively from a review of the literature [14,15,16,17,18,19,27,28,29]. Socioeconomic position (maternal education) and demography (maternal age, parity, history of fetal loss) were extracted from the baseline survey. Maternal education was categorized as college or lower and university or higher. Maternal age was under 25 and 25+ years. Parity was represented by a binary variable: primiparous and multiparous. History of fetal loss included yes or no.

Details of the other four potential covariates were obtained from telephone interviews after delivery, and included the number of ANC, delivery hospital, sex of the newborn, and birth weight. The number of ANC was divided into two groups, less than eight and at least eight times, as per the WHO recommendations for having a positive pregnancy experience [30]. Birth weights were divided into three groups: underweight (less than 2500 G), normal (2500–4000 G), and macrosomia (over 4000 G).

### 2.4. Statistical Analysis

All data were analyzed using the Statistical Package for Social Science (SPSS, IBM Corp., Released 2017. IBM SPSS Statistics for Windows, Version 25.0. Armonk, NY, USA: IBM Corp.). Descriptive statistics (frequencies, and percentages) were used to characterize the sample and calculate the prevalence of cesarean delivery. A chi-squared test (or Fisher’s exact test if applicable) was performed to assess the association of the independent variables with the mode of delivery (vaginal, cesarean section for medical reasons, and nonmedical reasons). The relationship between breastfeeding exclusively until 6 months and cesarean delivery for nonmedical reasons was assessed by a multivariable multinomial logistic regression model using a backward stepwise elimination approach, with adjustment of independent variables with a *p*-value < 0.25 in the bivariate analysis [31]. The results were presented as crude odds ratio (cOR) and adjusted odds ratio (aOR) along with 95% confidence intervals.

### 2.5. Ethical Approval

The study received ethical approval from the ethics committee of the Hanoi University of Public Health (Ref: 28/2019/YTCC-HD3) and the Curtin Human Research Ethics Committee (Ref: HRE2019-0143-03). Approval from relevant hospital authorities was obtained before accessing their documents, and written informed consent was taken from all mothers interviewed. Mothers understood that they have the freedom of withdrawal from the study at any time, without giving any reason, and without any negative consequences.

## 3. Results

### 3.1. Reasons for Having Cesarean Section Delivery

Of 554 mothers, 274 (49.5%, 95% CI: 45.3–53.6%) underwent cesarean section. The reasons for having a cesarean section delivery are presented in Table 1. Of all cesarean deliveries, 60.4% (95% CI: 54.4–66.0%) were for medical reasons, 30.8% (95% CI: 25.2–36.1%) for nonmedical reasons, and 9.1% (95% CI: 5.7–12.5%) unknown.

Regarding medical reasons, based on ICD-10 classification, “previous Cesarean section” was the most common indication (23.1%). Other indications included “prolonged and obstructed labor” at 9.2%, “the disorder of amniotic fluid” at 9.2%, “mal presentation” at 6.6%, “fetal distress” at 4.8%, “post-dated pregnancy” at 1.8%, “hypertensive disorder” at 1.5%, “antepartum haemorrhage including placenta praevia amniotic fluid disorder” at 1.1%, and other, 3.3%.

Nonmedical reasons included family’s preference—15.4%, doctor’s suggestion—12.5%, fear of vaginal delivery pain—1.5%, and more convenience in choosing the birth date—1.5%.

### 3.2. Characteristics of Mothers by Mode of Delivery

The differences between mode of delivery (vaginal, cesarean section for medical reasons or nonmedical ones) by other covariates are presented in Table 2. Mothers who did not know the reasons for having a cesarean section were excluded from the analysis.

Of 529 mothers included in the analysis, 71.1% were aged 25 years or over, and 58.8% obtained a university or higher degree. Half of the mothers (53.7%) were primiparous, and 11% had experienced fetal loss in earlier pregnancies. Of these, about 70% had at least eight ANC contacts, and 77.7% delivered in Hanoi Gynecology and Obstetrics Hospital.

Of the births, male sex accounted for 55.6%. Low birth weight (= or <2500 G) was found in 4.5%, and macrosomia (= or >4500 G) was found in 2.8%.

The statistically significant differences between potential covariables by mode of delivery (vaginal, cesarean section for medical reasons, and cesarean section for nonmedical reasons) were assessed by the *p*-value of a chi-squared test (or Fisher’s exact test if applicable) less than 0.05. Having cesarean section for medical reasons and vaginal delivery were found among mothers who had at least 8 antenatal contacts, and low birth weight or macrosomia (*p* < 0.05). Among mothers who had cesarean section for medical reasons, the proportion of those having least 8 antenatal contacts (39.0%) was higher than having fewer (11.8%); those who had a child with normal birth weight (29.4%) were lower than those had a child with low birth weight (41.7%) or macrosomia (73.3%). Among mothers who had cesarean section for nonmedical reasons, those who had at least 8 antenatal contacts (11.1%) was lower than those with fewer contacts (27.6%); those who had a child in normal birth weight (15.7%) were lower than those had a child with low birth weight (20.8%) but higher than those with a child with macrosomia (13.3%).

### 3.3. Exclusive Breastfeeding Intention and Cesarean Section

**Figure 1:**
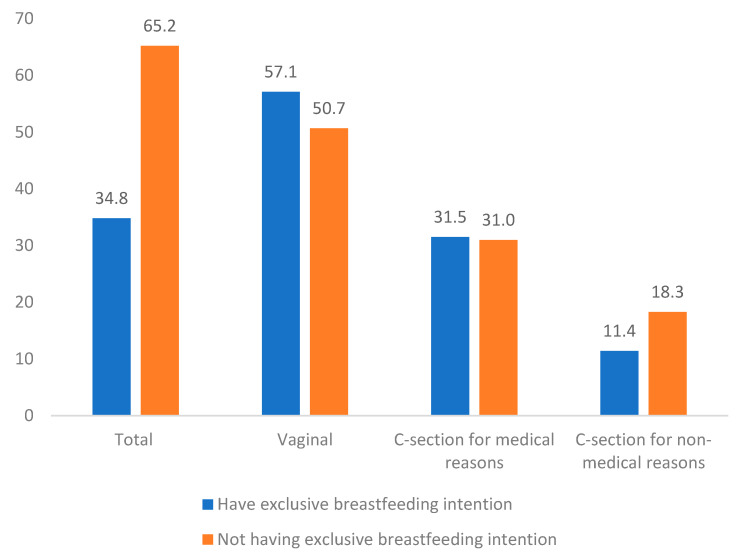
The exclusive breastfeeding intention and modes of delivery in Hanoi 2021 (*N* = 529).

In total, 34.8% of mothers had the prenatal intent to breastfeed exclusively until 6 months. The highest percentage of breastfeeding intention was found among vaginal delivery (57.1%) and lowest among those who had cesarean section for nonmedical reasons (11.4%) (Figure 1). The differences between having exclusive breastfeeding intention and delivery mode, 57.1% vs. 50.1% among vaginal delivery, 31.5% vs. 31.0% among cesarean delivery for medical reasons, and 11.4% vs. 18.3% nonmedical reasons, respectively, were not significant at a threshold of 0.05 (*p* = 0.107).

Initially, a multinomial logistic regression model was performed between intent to breastfeed exclusively until 6 months and having cesarean delivery for medical and nonmedical reasons. After that, the association was adjusted for the covariables with *p* < 0.25 in Table 2 which included maternal age, parity, having at least eight ANC, hospital of delivery, and birth weight. Results are presented in Table 3. Compared to those mothers who did not have an intention of exclusively breastfeeding until 6 months, the odds of those having the same intention for having “cesarean section for non-medical reasons (versus vaginal delivery)” were reduced by 44% on average (crude OR = 0.55, 95% CI: 0.31–0.96, *p* = 0.036). After adjusting for the covariables mentioned above, compared to those mothers who did not have an intention of exclusively breastfeeding until 6 months, the odds of those having the same intention for having “cesarean section for non-medical reasons (versus vaginal delivery)” were reduced by 45% average, (adjusted OR = 0.55, 95% CI: 0.31–0.96, *p* = 0.034). The differences between “cesarean section for medical reasons (versus vaginal delivery)” and breastfeeding intention were not statistically significant in both models.

## 4. Discussion

Our study examined the relationship between intent to breastfeed exclusively until 6 months and having a cesarean section for nonmedical reasons among single-delivery women in Hanoi, Vietnam. The cesarean section rate was 49.5%, which is similar to reports from the Hanoi Obstetrics and Gynecology Hospital and other public hospitals in urban areas in Vietnam [14]. The rate of cesarean section increased from 2.6% to 10.1% in the period 1999–2010 [19], and reached up to about 50% in Hanoi 2020.

About 80% of our participants delivered in the Hanoi Obstetrics and Gynecology Hospital, a specialist hospital for obstetrics and gynecology, where the cesarean section rates could be overestimated compared to the general population. However, we found 30.8% of mothers had cesarean section for nonmedical reasons (doctors’ suggestions or family’s preference). A previous qualitative study in Vietnam suggested that psychological fear occurred among women and families, and doctors were the main determinants for driving the requests for cesarean section [32]. In China, a country with high rates of cesarean section, it has been suggested that doctors preferred cesarean sections because of financial drivers and malpractice fears [12]. The latter concern is unfounded since operative delivery carries a higher risk.

One in every three mothers had cesarean section due to family preference, and we did not differentiate between the woman’s preference herself and other family members as it is out of the scope of this study. Despite having a vaginal delivery preference, women in Vietnam still underwent cesarean delivery because of the support and perceived wishes of parents and peers [33]. Previous studies in other countries suggested that mothers’ perceived pain, maternal short-term risks, and newborn risks of vaginal delivery while opting for a sense of control over the birth, and diminished feelings of fear from cesarean section [12,13]. A more convenient ability to choose an auspicious date for birth was reported in about 1.5% of mothers, which is similar to results of previous qualitative studies in Vietnam [32], and in China [12]. Fear of vaginal delivery pain was the most common reason for cesarean section Fear of vaginal delivery pain Fear of vaginal delivery pain Fear of vaginal delivery pain Fear of vaginal delivery pain [12], but in our study, this was only the case for 1.5% of mothers. The reason behind family preference should be investigated in further studies.

We found maternal age and parity were associated with cesarean section, confirming the results of previous studies [17,34]. Mothers were more likely to have cesarean section at higher parity, mostly because of prior cesarean section. Prior cesarean section was the most common reason for having a repeated one, as we found in this study and other studies in Vietnam [15,18], and other countries [34,35]. Vaginal delivery after cesarean is a reasonable and safe choice for the majority of women with prior cesarean [36]. However, prior cesarean section is an indicator for conducting cesarean section in technical guidelines for obstetrics in Vietnam [37].

The Vietnam Ministry of Health has recommended since 2016 that mothers have at least four antenatal visits following the WHO guidelines for healthy pregnant women in a limited-resource setting, to prevent maternal and neonatal adverse outcomes. In urban areas such as Hanoi, about 70% of our study participants had at least eight antenatal contacts according to the WHO guidelines for healthy pregnant women targeting positive pregnancy experience [30]. An analysis of six developing countries in Latin America and South Asia, including Vietnam, showed that women with a higher socioeconomic background and better access to antenatal services are the most likely to undergo a cesarean section [29]. We also found that compared with vaginal delivery, women who had at least eight antenatal contacts were more likely to have a medical cesarean delivery than those who had fewer. The likelihood of having a risky pregnancy is associated with a higher number of antenatal contacts and having a cesarean section, but this may be because “at-risk” mothers are requested to come in more frequently. The association between a higher level of women’s empowerment, number of antenatal contacts, and having a cesarean delivery was also reported in Pakistan [34]. On the other hand, mothers who had at least eight antenatal contacts were less likely to have cesarean delivery for nonmedical reasons. Therefore, controlling the effect of antenatal contacts allows us to better distinguish between the medical and nonmedical reasons for cesarean delivery.

We found mothers who had an intention to breastfeed exclusively until 6 months were significantly less likely to have cesarean delivery for nonmedical reasons. The reduction in all cesarean sections for mothers who intended to breastfeed shows that the reduction in the cesarean section for nonmedical reasons was not achieved by just shifting categories. This is a similar result to a previous study in Alberta, which suggested that women who had a planned cesarean section were less likely to have intent to breastfeed [8]. However, a prospective cohort study conducted in the United Kingdom, where breastfeeding rates are very low, found that the intended breastfeeding predelivery was not associated with having a cesarean section [21]. Other studies in the United Kingdom or Canada, for example, reported both breastfeeding intentions and cesarean section, but unfortunately, their relationship was not examined [38,39].

While one in every three mothers had a cesarean section due to family preference in our study, similar to the proportion found in China [12], the nonclinical interventions for reducing this unnecessary surgery mostly target healthcare professionals [40]. Most effective interventions included implementation of guidelines combined with mandatory second opinion or audits in high-income countries. In low- and middle-income countries, a prenatal education program that emphasizes the natural process and benefits of vaginal delivery and breastfeeding to health outcomes of both mothers and children in the short term and long term could reduce the preference for having a cesarean section. Further studies need to be conducted to confirm the relationship between intention to breastfeed and having a cesarean delivery.

Several limitations need to be considered when interpreting the results of this study. First, all participants involved in the project had a smartphone. More than 85% of the population own a smartphone in urban areas in Vietnam and this could have biased the sample slightly towards the middle and upper socioeconomic groups. Secondly, participants who agreed to be involved in the projects may have more knowledge and concern about maternal and child health care than the general population. While the follow-up rate was high (85.9%) the missing data could have introduced some bias into the results. The data was based on the responses of mothers, although the possibility of them not knowing they had a cesarean section would be remote. The cesarean section rate in this study was comparable to hospitals’ reports. This paper reports results from the control group to minimize bias from the effect of interventions on the mode of delivery, if any. The sample size is therefore smaller and may have produced wider confidence limits. The cesarean section rates may not be representative of all of the mothers in Hanoi.

## 5. Conclusions

In Vietnam, one in every three cesarean sections was for nonmedical reasons. This study adds to the growing evidence that there is overuse of cesarean section due to family and health-professional preference reasons in urban Vietnam. Mothers who intended to breastfeed had a lower rate of cesarean section. Education on breastfeeding and the promotion of preparation for exclusive breastfeeding may have the potential to reduce the rate of cesarean sections. Further studies in different areas of Vietnam are needed to confirm the relationship between breastfeeding intentions and reduction in cesarean sections. A clinical trial of breastfeeding promotion as a possible intervention for reducing cesarean sections and improving maternal and child health would be worthwhile.

## Figures and Tables

**Table 1:** The main reason for having cesarean delivery (*n* = 274).

Reasons	*n*	%
**Medical Reasons**	**165**	**60.4**
Previous cesarean delivery	63	23.1
Prolonged & obstructed labor	25	9.2
Disorder of amniotic fluid	25	9.2
Mal presentation	18	6.6
Fetal distress	13	4.8
Post-dated pregnancy	5	1.8
Hypertensive disorder	4	1.5
Antepartum hemorrhage	3	1.1
Other medical reasons (diabetes, accidence, etc.)	9	3.3
**Nonmedical reasons**	**84**	**30.8**
Doctor’s suggestion	34	12.5
Family’s preference	42	15.4
Fear of vaginal delivery pain	4	1.5
More convenient in choosing the birth date	4	1.5
**Unknown**	**25**	**9.1**

**Table 2:** Characteristics of mothers and infants by mode of delivery in Hanoi 2021 (*N* = 529).

Characteristics	*N* (%)	Vaginal *n*, %	C-Section for Medical Reasons *n*, %	C-Section for Nonmedical Reasons *n*, %	*p*
Maternal age (years)					0.061
<25	153 (28.9)	93 (60.8)	38 (24.8)	22 (14.4)	
>=25	376 (71.1)	187 (49.7)	127 (33.8)	62 (16.5)	
Education					0.410
College or lower	218 (41.2)	110 (50.5)	75 (34.4)	33 (13.1)	
University or higher	311 (58.8)	170 (54.7)	90 (28.9)	51 (16.4)	
Parity					0.093
Primiparous	284 (53.7)	162 (57.0)	78 (27.5)	44 (15.5)	
Multiparous	245 (46.3)	118 (48.2)	87 (35.5)	40 (16.3)	
History of fetal loss					0.422
No	471 (89.0)	254 (53.9)	144 (30.6)	73 (15.5)	
Yes	58 (11.0)	26 (44.8)	21 (36.2)	11 (19.0)	
Having at least 8 antenatal contacts					
No	152 (28.7)	92 (60.5)	18 (11.8)	42 (27.6)	**<0.0001**
Yes	377 (71.3)	188 (49.9)	147 (39.0)	42 (11.1)	
Hospital of delivery					0.107
Other hospitals	118 (22.3)	63 (53.4)	43 (36.4)	12 (10.2)	
Hanoi Gynecology and Obstetrics Hospital	441 (77.7)	217 (52.8)	122 (29.7)	72 (17.5)	
Child’s sex					0.447
Boy	294 (55.6)	149 (50.7)	98 (33.3)	47 (16.0)	
Girl	235 (44.4)	131 (55.7)	67 (28.5)	37 (15.7)	
Birth weight (gram)					**0.003**
<2500	24 (4.5)	9 (37.5)	10 (41.7)	5 (20.8)	
2500–4000	490 (92.6)	269 (54.9)	144 (29.4)	77 (15.7)	
>4000	15 (2.8)	2 (13.3)	11 (73.3)	2 (13.3)	

**Table 3:** Association between breastfeeding intentions and having a cesarean section.

Characteristics	Crude OR (95% CI)	*p* ^a^	Adjusted OR (95% CI)	*p* ^b^
Mode of delivery				
Vaginal delivery	1		**1**	
C-section for nonmedical reasons	**0.56 (0.32–0.96)**	**0.036**	**0.55 (0.31–0.96)**	**0.034**
C-section for medical reasons	0.62 (0.34–1.11)	0.105	0.64 (0.35–1.18)	0.154

^a^ From the multinomial logistic regression model between breastfeeding intentions and having cesarean section. ^b^ Adjusted for maternal age, parity, having at least eight ANC, hospital of delivery, and birth weight. CI: confidence interval; OR: odds ratio.

## Data Availability

The database used for this study is part of an ongoing trial in Hanoi, Vietnam. The data can be assessed by contacting Colin Binns (c.binns@curtin.edu.au) who is the PI of the project.
